# SIRT6 overexpression retards renal interstitial fibrosis through targeting HIPK2 in chronic kidney disease

**DOI:** 10.3389/fphar.2022.1007168

**Published:** 2022-09-12

**Authors:** Xiaoxue Li, Wenxin Li, Zhengzhipeng Zhang, Weidong Wang, Hui Huang

**Affiliations:** ^1^ Department of Cardiology, The Eighth Affiliated Hospital, Sun Yat-sen University, Shenzhen, China; ^2^ Department of Pathophysiology, Zhongshan School of Medicine, Sun Yat-sen University, Guangzhou, China

**Keywords:** SIRT6, HIPK2, renal interstitial fibrosis, chronic kidney disease, vitamin B3, renal function

## Abstract

**Introduction:** Renal interstitial fibrosis is a common pathophysiological change in the chronic kidney disease (CKD). Nicotinamide adenine dinucleotide (NAD)-dependent deacetylase sirtuin 6 (SIRT6) is demonstrated to protect against kidney injury. Vitamin B3 is the mostly used form of NAD precursors. However, the role of SIRT6 overexpression in renal interstitial fibrosis of CKD and the association between dietary vitamin B3 intake and renal function remain to be elucidated.

**Methods:** Wild-type (WT) and SIRT6-transgene (SIRT6-Tg) mice were given with high-adenine diets to establish CKD model. HK2 cells were exposed to transforming growth factor β1 (TGF-β1) *in vitro* to explore related mechanism. Population data from Multi-Ethnic Study of Atherosclerosis (MESA) was used to examine the association between dietary vitamin B3 intake and renal function decline.

**Results:** Compared to WT mice, SIRT6-Tg mice exhibited alleviated renal interstitial fibrosis as evidenced by reduced collagen deposit, collagen I and α-smooth muscle actin expression. Renal function was also improved in SIRT6-Tg mice. Homeodomain interacting protein kinase 2 (HIPK2) was induced during the fibrogenesis in CKD, while HIPK2 was downregulated after SIRT6 overexpression. Further assay *in vitro* confirmed that SIRT6 depletion exacerbated epithelial-to-mesenchymal transition of HK2 cells, which might be linked with HIPK2 upregulation. HIPK2 was inhibited by SIRT6 in the post-transcriptional level. Population study indicated that higher dietary vitamin B3 intake was independently correlated with a lower risk of estimate glomerular filtration rate decline in those ≥65 years old during follow-up.

**Conclusion:** SIRT6/HIPK2 axis serves as a promising target of renal interstitial fibrosis in CKD. Dietary vitamin B3 intake is beneficial for renal function in the old people.

## Introduction

Chronic kidney disease (CKD) is highly prevalent in the world, especially in those with diabetes, hypertension as well as the old population (Carney, 2020; Collaboration, 2020). Although the initial etiology varies in CKD patients, renal interstitial fibrosis is the common and final pathological change (Ruiz-Ortega et al., 2020). Renal interstitial fibrosis is characterized by abnormal accumulation of extracellular matrix, leading to progression to end-stage renal disease (ESRD) (Livingston et al., 2016; Luo et al., 2018). Notably, tubular epithelial cells are of large plasticity, which undergo epithelial-to-mesenchymal transition (EMT) in response to renal injury. Tubular epithelial cells increasingly express α-smooth muscle actin (αSMA) and produce collagen to potentiate fibrotic pathogenesis in CKD (Liu et al., 2019; Bozic et al., 2020).

Sirtuin 6 (SIRT6) belongs to the conserved nicotinamide adenine dinucleotide (NAD^+^)-dependent chromatin deacetylases family, which is implicated in genomic stability, inflammation and energy metabolism regulation (Chang et al., 2020). Overexpressed SIRT6 is demonstrated to protect against acute kidney injury (Li et al., 2018; Zhang et al., 2019). SIRT6 also impedes diabetic nephropathy by mitigating podocyte and proximal tubules injury (Huang et al., 2017; Muraoka et al., 2019; Yang et al., 2020). Hyperactivation of transforming growth factor β (TGF-β) signaling is the master inducer of fibrosis pathogenesis (Meng et al., 2016). SIRT6 deficiency significantly activates TGF-β signaling and exacerbates aging-related organ fibrosis (Maity et al., 2020). Meanwhile, knockdown of SIRT6 aggravates unilateral ureteral obstruction-induced renal fibrosis (Cai et al., 2020). As previous study shows that SIRT6 is upregulated during renal injury (Cai et al., 2020), whether further SIRT6 overexpression is capable of blocking CKD-associated renal interstitial fibrosis remains to be elucidated.

Homeodomain interacting protein kinase 2 (HIPK2) is a nuclear serine/threonine kinase and initially discovered as a corepressor of NK homeoproteins (Choi et al., 2013). Recently, HIPK2 is regarded as a central contributor to renal fibrosis and accounts for multiple pro-fibrosis signaling pathways including TGF-β, Wnt/β-catenin and Notch pathway (Jin et al., 2012; Xiao et al., 2020). Inhibiting of HIPK2 also exhibits a remarkable alleviation in renal fibrosis (Liu et al., 2017; Xu et al., 2019). However, its potential regulation by SIRT6 is still undetermined.

NAD is a critical co-enzyme involved in cellular physiological function, which determines the activity of diverse enzymes including sirtuins, PARPs, CD38 and so on (Chini et al., 2021). Although activator of SIRT6 is unavailable in clinical, intervention in NAD metabolism might be an alternative strategy. In fact, emerging evidence suggests that NAD level declines in kidney injury, while NAD supplements was demonstrated to protect against kidney diseases (Katsyuba et al., 2018; Manrique-Caballero et al., 2021; Bignon et al., 2022). Supplements with different forms of vitamin B3, the NAD precursors, are also demonstrated to improve SIRT6 activity and expression (Diani-Moore et al., 2017; Hou et al., 2018; Kim et al., 2022; Ru et al., 2022). Several NAD precursors have been tested in clinical, and vitamin B3 as the simple and mostly use form of NAD precursor shows a promisingly positive result (Bagcchi, 2015; Dollerup et al., 2018; Pirinen et al., 2020). Even though, the effect of dietary vitamin B3 intake on renal function progression remains unknown.

In this study, we generated SIRT6-transgenic (SIRT6-Tg) mice to see the protective effect of SIRT6 overexpression in CKD-associated renal interstitial fibrosis. Upregulation of HIPK2 in fibrotic kidney and tubular epithelial cells undergoing EMT was also blocked after SIRT6 overexpression. Using population data from MESA, we found that higher dietary vitamin B3 intake is related to lower risk of renal function decline in the old people. Our finding suggested an involvement of SIRT6/HIPK2 axis in renal interstitial fibrosis and provided a promising target in CKD prevention.

## Materials and methods

### Study population

The population data was obtained from the Multi-Ethnic Study of Atherosclerosis (MESA) dataset. The detailed design and examination of MESA have been described previously (Bild et al., 2002). Briefly, MESA is a prospective observational cohort study, which recruited 6,814 participants between July 2000 and September 2002 from different races. In this study, we included participants with baseline dietary vitamin B3 intake and estimate glomerular filtration rate (eGFR) measurements at Exam1 (2000–2002) and follow-up eGFR at Exam3 (2004–2005). The study flowchart was presented in Supplementary Figure S1. Of the 6,814 participants, those with missing covariates (*n* = 220), missing dietary vitamin B3 and total energy intake data (*n* = 546) and missing follow-up eGFR data from Exam 3 (*n* = 789) were excluded. Adjusted dietary vitamin B3 intake was calculated as vitamin B3 per 1,000 kcal total energy intake per day. Renal function decline was defined as ≥10% drop in eGFR according to the baseline (Huang et al., 2021).

### Diet assessment

The usual diet of participants was characterized using a self-administered 120-item food frequency questionnaire (FFQ), which included the type and frequency of foods consumed over the past year. Participants were recorded with the serving size (small, medium or large) and frequency of consumption (average times per day, week or month), with corresponding weight estimated according to National Health and Nutrition Examination Survey data. Approximate amounts of dietary vitamin B3 intake was imputed with the DietSys Nutrient Analysis Program (Nettleton et al., 2006; Gao et al., 2021).

### Animal experiments

Eight-week-old C57BL/6J mice were obtained from the Laboratory Animal Center of Sun Yat-sen University. SIRT6-Tg mice with C57BL/6J background were generated and bred as previously reported (Li et al., 2022). The mice were randomly given a chow diet as the control group, or a high adenine diet (0.25% adenine and 1.2% phosphorus) for 12 weeks as the CKD-related renal fibrosis group. All mice were housed in a constant temperature and a 12 h light-dark cycle with free access to water and food. The kidneys were harvested for further research. The animal experiment was conducted according to the animal ethical standards and approved by the Ethics Committee of Zhongshan School of Medicine, Sun Yat-sen University.

### Cell culture

Human renal tubular epithelial cells line (HK2 cells) was purchased from the American Type Culture Collection and cultured in Dulbecco’s modified Eagle’s medium/F12 containing 10% fetal bovine serum (FBS) (Gibco). To induce cellular fibrotic model, TGF-β1 (R&D Systems) was added into the medium for 48 h. For SIRT6 depletion, HK2 cells were transfected with control small interfering RNA (siRNA) and SIRT6 siRNA (IGE Biotechnology, China) using Lipofectmaine RNAiMAX (Invitrogen) according to the manufacturer’s instructions. The sequence of SIRT6 siRNA was AGT​TCG​ACA​CCA​CCT​TTG​A (5′-3′).

### Western blot analysis

Cells or tissue homogenates were added with RIPA lysis buffer on ice for 15 min. The lysates were collected and centrifuged at 13000 rpm at 4°C for 20 min. Proteins were separated on sodium dodecyl sulfate polyacrylamide gel following with transferred to PVDF membranes (Millipore). The membranes were blocked with 5% skim milk and incubated with specified antibodies. Band intensity was analyzed with the ImageJ software.

Antibodies against α-SMA (ab21027), SIRT6 (ab191385) and HIPK2 (ab108543) were obtained from Abcam. Antibody against Tubulin (11224-1-AP) was obtained from Proteintech. Antibody against HIPK2 (sc-100383) was obtained from Santa Cruz Biotechnology. Antibodies against E-cadherin (3195S), collagen I (72026S), GAPDH (5174S), secondary anti-rabbit (4412S) and anti-mouse (4408S) were purchased from Cell Signaling Technology.

### Reverse transcription and real-time qPCR

Cellular mRNA was isolated from HK2 cells using RNAiso Plus reagent (TaKaRa, Japan) and then reverse-transcribed to cDNA according to the manufacturer’s instructions (TaKaRa, Japan). Gene expression was quantified with the real-time PCR system (Roche LightCycler 480) and detected by SYBR Green Premix (TaKaRa, Japan). Relative expression of mRNA was determined using 2^−ΔΔCT^ method with GAPDH as a reference gene.

### Hematoxylin and eosin, masson trichrome staining

Paraffin-embedded kidney sections were deparaffinized and stained according to the manufacturer’s protocols by using the Hematoxylin and eosin staining kit (Solarbio, China), and Masson trichrome staining kit (Solarbio, China). Images were captured with light microscopy (Nikon NiU).

### Immunohistochemical staining

Paraffin-embedded kidney sections were deparaffinized and rehydrated following by heat-mediated antigen retrieval with 10% citrate buffer. Endogenous peroxidase activity was eliminated with 3% H_2_O_2_. Then the sections were incubated with primary antibodies overnight at 4°C, covered with corresponding secondary antibodies in the room temperature and visualized with diaminobenzidine. Images were captured with light microscopy (Nikon NiU).

### Immunofluorescence staining

HK2 cells were fixed with 4% paraformaldehyde for 15 min and permeabilized with 0.1% Triton X-100 for 10 min. After blocking, cells were incubated with primary antibiotics overnight and then detected with fluorescent dye-conjugated secondary antibody. Nuclei were stained with DAPI for 10 min. Images were captured with fluorescence microscope (Olympus IX83).

### Statistical analysis

All data were analyzed with SPSS and GraphPad Prism 8.0 software. The results were presented as mean ± S.E.M. To compare two group, Student’s *t* test or nonparametric Mann-Whitney *U* test were performed, while ordinary one-way ANOVA was conducted to compare multiple groups. A logistic regression model was established to compute risk ratios (RRs) and 95% confidence intervals (CI) to assess the association between dietary niacin intake and risk of renal function decline. *p* value < 0.05 was considered statistically significant.

## Results

### SIRT6 overexpression impedes renal interstitial fibrosis and renal function deterioration in chronic kidney disease

**FIGURE 1:**
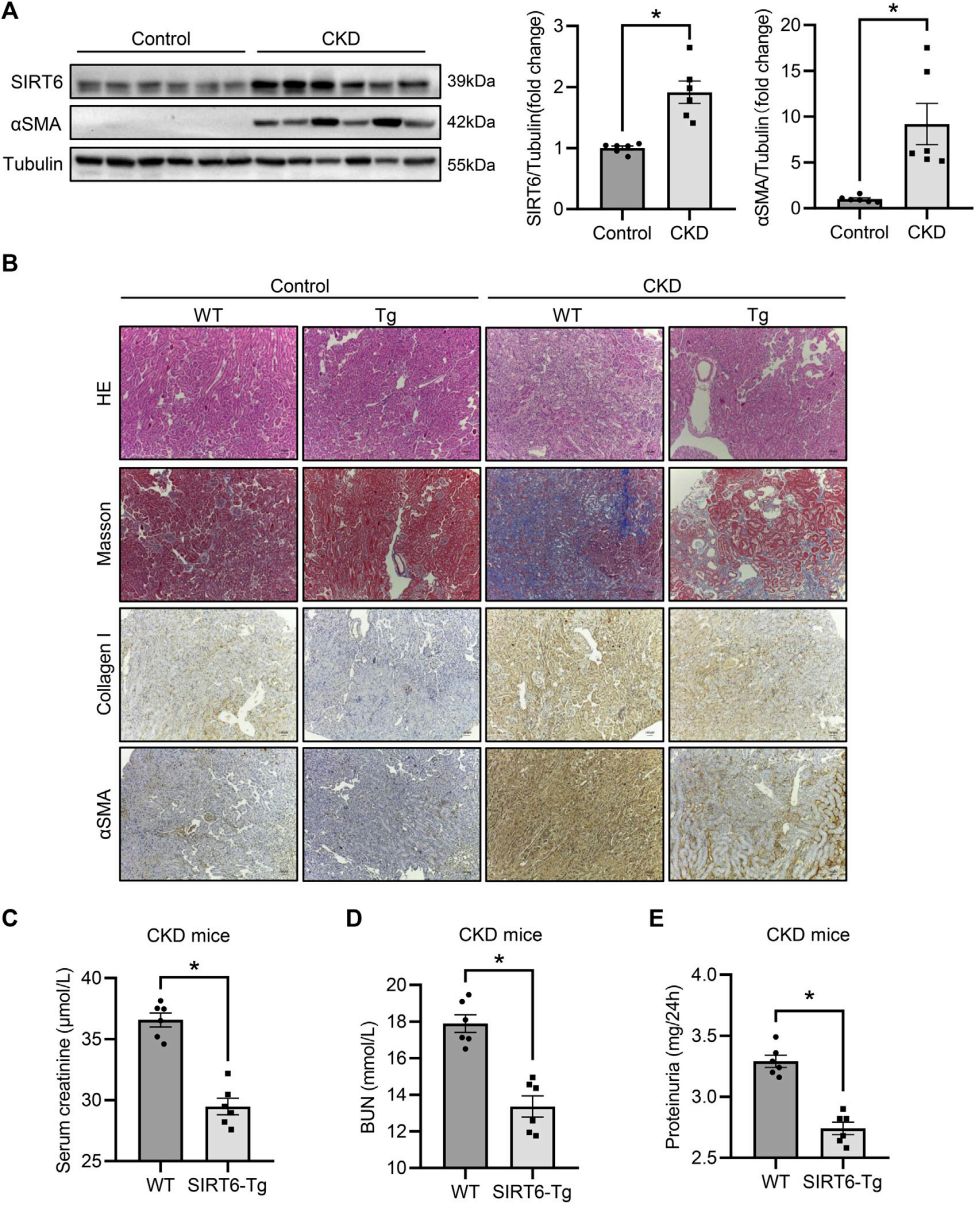
SIRT6 overexpression protected against renal interstitial fibrosis. **(A)** Western blot analysis of SIRT6 and αSMA expression in fibrotic kidneys of CKD mice. *n* = 6. **(B)** Representative hematoxylin and eosin staining, Masson trichrome staining and immunohistochemical images of collagen I and αSMA expression in kidneys. Scale bar, 100 μm. **(C–E)** Sera from WT and SIRT6-Tg of CKD mice were tested for serum creatinine, BUN and proteinuria for 24 h *n* = 6. All values are presented as mean ± SEM. *p < 0.05.

To verify the effect of SIRT6 in renal interstitial fibrosis, high adenine diet-induced CKD model was established in WT and SIRT6-Tg mice. As expected, αSMA was produced in the kidney of CKD, accompanied by elevated SIRT6 expression (Figure 1A). In WT mice with CKD, hematoxylin and eosin staining confirmed an abroad mononuclear cells infiltration and kidney tubules damage, while Masson trichrome staining showed the collagen deposition, all of which were alleviated in SIRT6-Tg mice (Figure 1B). A significant upregulation of collagen I and αSMA in CKD was evidenced by immunohistochemistry staining. Such effect was also less apparent in SIRT6-Tg mice (Figure 1B). Additionally, the results of renal function assessments confirmed that serum creatinine, blood urea nitrogen (BUN) and proteinuria for 24 h were improved in SIRT6-Tg mice (Figures 1C–E). Taken together, these data reveal that SIRT6 overexpression protects against renal interstitial fibrosis in CKD.

### Upregulation of HIPK2 in chronic kidney disease is blocked by SIRT6 overexpression

**FIGURE 2:**
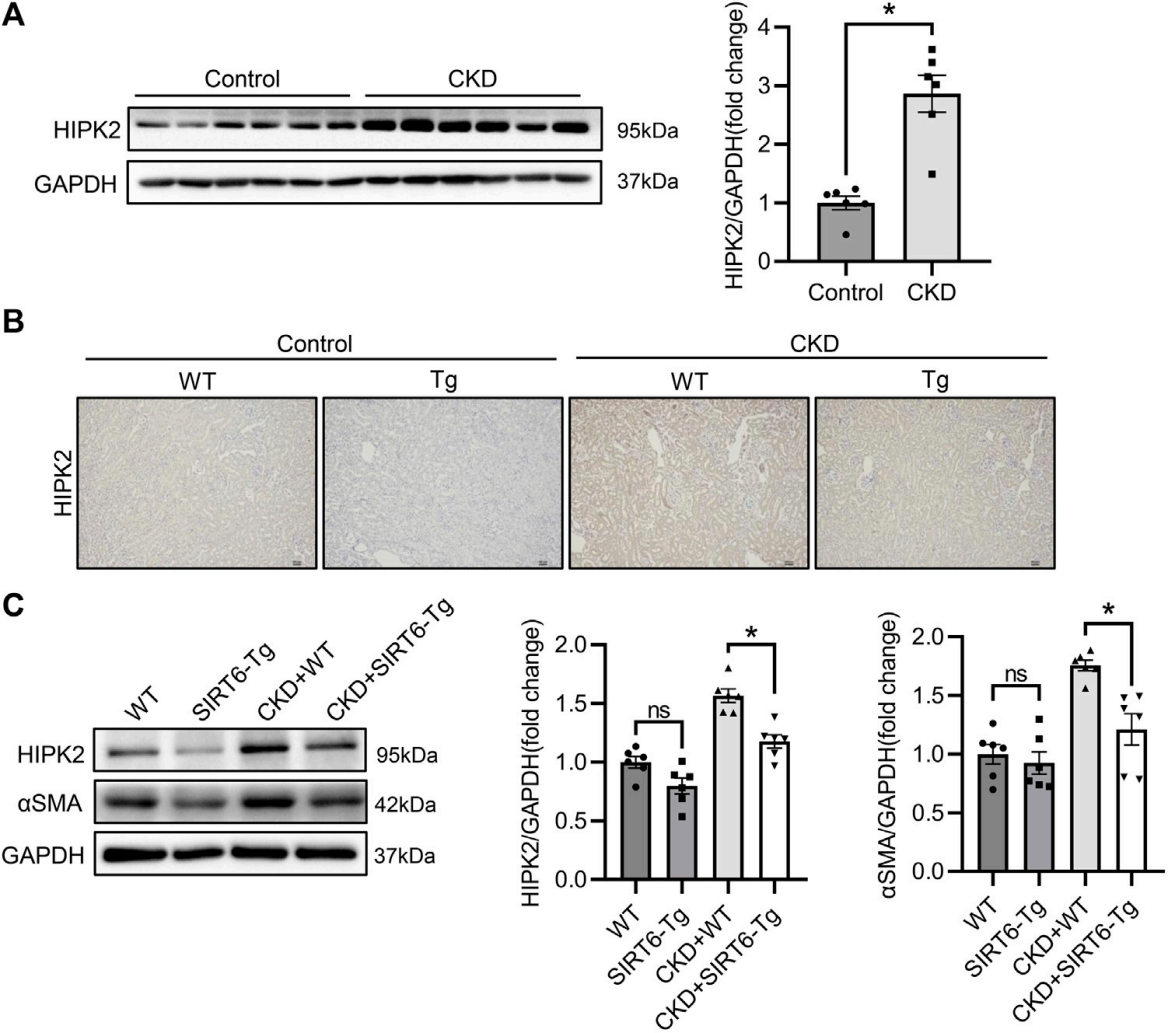
HIPK2 was downregulated in the kidneys of SIRT6-Tg mice. **(A)** Western blot analysis of HIPK2 expression in fibrotic kidneys of CKD mice. *n* = 6. **(B)** Representative immunohistochemical images of HIPK2 in kidneys of SIRT6-Tg mice. Scale bar, 100 μm. **(C)** Western blot analysis of HIPK2 and αSMA expression in fibrotic kidneys of WT and SIRT6-Tg mice. *n* = 6. All values are presented as mean ± SEM. *p < 0.05.

Considering the key modulation of HIPK2 in pro-fibrosis signaling, we next detect the expression of HIPK2 in CKD. As expected, HIPK2 was largely induced in the fibrotic kidney of CKD (Figure 2A). Since SIRT6 overexpression exhibited a prominent mitigation in renal interstitial fibrosis, we speculated that such effect might be attributed to HIPK2 downregulation. Notably, compared to WT mice, SIRT6-Tg mice presented a reduced HIPK2 expression both in kidneys of control and CKD mice, as evidenced by immunohistochemical and western blot analysis (Figures 2B,C). These findings suggest that the beneficial role of SIRT6 in renal interstitial fibrosis prevention might be associated with HIPK2 signaling inhibition.

### SIRT6 and HIPK2 are induced by TGF-β1 in renal tubular epithelial cells

**FIGURE 3:**
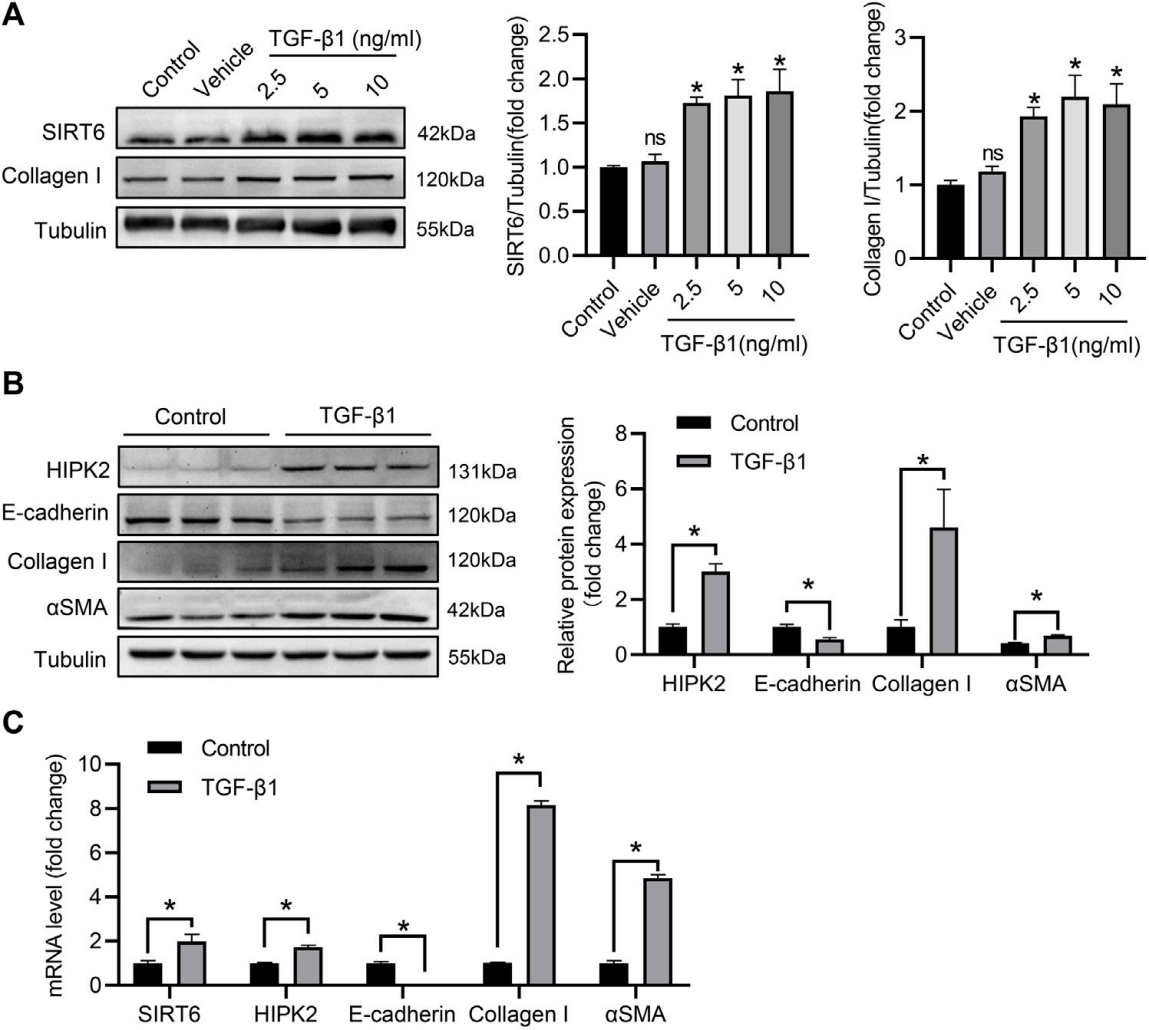
SIRT6 and HIPK2 are induced by TGF-β1 in HK2 cells. **(A)** Western blot analysis of SIRT6 and collagen I expression in HK2 cells exposed to TGF-β1 for 48 h with indicated dose. *n* = 3. **(B)** Western blot analysis of HIPK2 expression in HK2 cells exposed to 2.5 ng/ml TGF-β1 for 48 h *n* = 3. **(C)** Quantitative real-time PCR analysis of genes expression in HK2 cells treated with TGF-β1. *n* = 3. All values are presented as mean ± SEM. *p < 0.05.

To further elucidate the modulation of SIRT6 and HIPK2 in renal interstitial fibrosis, HK2 cells were treated with TGF-β1 to establish fibrotic model *in vitro*. As shown in Figure 3A, SIRT6 was upregulation in HK2 cells after TGF-β1 treatment for 48 h in a dose-dependent manner, together with increased collagen I expression. Consistently, HK2 cells switched from epithelial to mesenchymal phenotype, with the evidence of reduced epithelial marker E-cadherin and higher αSMA and collagen I expression. HIPK2 was also significantly elevated after TGF-β1 exposure (Figure 3B). Furthermore, quantitative real-time PCR analysis confirmed the phenotypic transition of HK2 cells, and both SIRT6 and HIPK2 were induced by TGF-β1 in transcriptional level (Figure 3C). Collectively, SIRT6 and HIPK2 are upregulated by TGF-β1 in renal tubular epithelial cells undergoing EMT.

### SIRT6 inhibits HIPK2 signaling by post-transcriptional regulation

**FIGURE 4:**
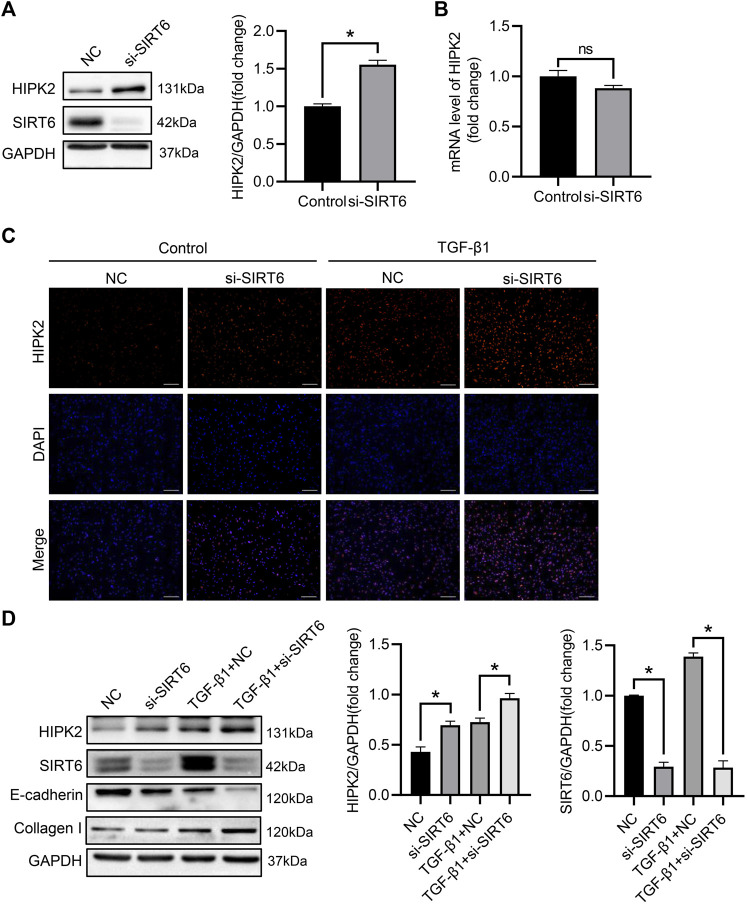
HIPK2 was downregulated by SIRT6 in the post-transcriptional level. **(A)** Western blot analysis of SIRT6 and HIPK2 expression after indicated transfection. *n* = 3. **(B)** Quantitative real-time PCR analysis of HIPK2 expression after SIRT6 depletion. *n* = 3. **(C)** Representative immunofluorescence images of HIPK2 in TGF-β1-induced HK2 cells after SIRT6 depletion. Scale bar, 200 μm. **(D)** Western blot analysis of SIRT6 and HIPK2 expression in TGF-β1-induced HK2 cells after SIRT6 depletion. *n* = 3. All values are presented as mean ± SEM. *p < 0.05.

Accumulating study suggest that the protein level of HIPK2 is determined by the post-transcriptional modification (de la Vega et al., 2012; Wook Choi and Yong Choi, 2014). Deacetylation of HIPK2 by deacetylases subsequently leads to its proteasomal degradation (Hwang et al., 2013). To explore the potential regulation of SIRT6 in HIPK2, small intervening RNA was utilized to inhibit SIRT6 expression in HK2 cells. As expected, the expression of SIRT6 was largely blocked after intervention, while HIPK2 was upregulated oppositely in the same time (Figure 4A). However, SIRT6 deficiency showed no impact on the mRNA level of HIPK2 (Figure 4B), indicating that HIPK2 was regulated by SIRT6 in post-transcriptional manner. Immunofluorescence also confirmed that the induction of HIPK2 by TGF-β1 exposure was exacerbated after SIRT6 depletion (Figure 4C). SIRT6 depletion promoted the phenotypic transition of HK2 cells, accompanied by HIPK2 upregulation (Figure 4D). Together, these data suggest a post-transcriptional downregulation of HIPK2 by SIRT6.

### Association between dietary vitamin B3 intake and renal function decline

Although the protective role of SIRT6 in CKD is prospective, drug target at SIRT6 is still unavailable in clinical. Indeed, the activity of SIRT6 is determined by the level of NAD. Since vitamin B3 is the mostly used form of NAD precursors, it propelled us to examine the association between dietary vitamin B3 intake and renal function progression. A total of 5,259 participants with a mean age of 61.8 ± 9.8 years old and 47.3% men from MESA was included in our study. As presented in Table 1, those with higher dietary vitamin B3 intake are prone to be Chinese American, diabetes, having higher fasting plasma glucose, less smoker and drinker, lower body mass index, total cholesterol, low-density lipoprotein cholesterol and homocysteine (*p* < 0.05). There was no significant difference in age, baseline eGFR, serum creatinine, hypertension prevalence, blood pressure, triglycerides, high-density lipoprotein cholesterol, use of angiotensin receptor blockers and angiotensin-converting enzyme inhibitors among the four groups.

**TABLE 1:** Baseline characteristics by the quartiles of energy-adjusted dietary vitamin B3 intake.

Characteristics	Total (*n* = 5,259)	Quartile 1 (*n* = 1,315)	Quartile 2 (*n* = 1,315)	Quartile 3 (*n* = 1,315)	Quartile 4 (*n* = 1,314)	*p* value
Adjusted dietary vitamin B3 intake (mg/day)	11.0 ± 2.8	7.9 ± 1.0	10.0 ± 0.4	11.5 ± 0.5	14.7 ± 2.6	<0.001
Baseline eGFR (mL/min/1.73m2)	78.1 ± 15.8	77.7 ± 16.0	77.9 ± 15.6	78.5 ± 15.4	78.3 ± 16.1	0.540
Serum creatinine (mg/dl)	0.9 ± 0.2	1.0 ± 0.2	1.0 ± 0.2	0.9 ± 0.2	0.9 ± 0.2	0.322
Age	61.8 ± 9.8	62.1 ± 10.4	61.7 ± 10.2	61.6 ± 10.0	61.8 ± 9.8	0.572
Male [n (%)]	2,488 (47.3%)	614 (46.7%)	644 (49.4%)	649 (49.4%)	581 (44.2%)	0.031
Race [n (%)]						<0.001
White	2,168 (41.2%)	545 (41.4%)	573 (43.6%)	499 (37.9%)	551 (41.9%)	
Black	1,324 (25.2%)	336 (25.6%)	332 (25.2%)	326 (24.8%)	330 (25.1%)	
Hispanic	1,105 (21.0%)	370 (28.1%)	319 (24.3%)	253 (19.2%)	163 (12.4%)	
Chinese American	662 (12.6%)	64 (4.9%)	91 (6.9%)	237 (18.0%)	270 (20.5%)	
Hypertension [n (%)]	2,268 (43.1%)	575 (43.7%)	553 (42.1%)	571 (43.4%)	569 (43.3%)	0.832
Diabetes [n (%)]	588 (11.2%)	117 (8.9%)	129 (9.8%)	162 (12.3%)	180 (13.7%)	<0.001
Smoking status [n (%)]						<0.001
Never	2,697 (51.3%)	635 (48.3%)	654 (49.7%0	704 (53.5%)	704 (53.6%0	
Former	1951 (37.1%)	484 (36.8%)	506 (38.5%)	473 (36.0%)	488 (37.1%)	
Current	611 (11.6%)	196 (14.9%)	155 (11.8%)	138 (10.5%)	122 (9.3%)	
Drinking status [n (%)]						<0.001
Never	1,064 (20.2%)	250 (19.0%)	234 (17.8%)	261 (19.8%)	319 (24.3%)	
Former	1,177 (22.4%)	335 (25.5%)	272 (20.7%)	293 (22.3%)	277 (21.1%)	
Current	3,018 (57.4%)	730 (55.5%)	809 (61.5%)	761 (57.9%)	718 (54.6%)	
BMI (kg/m^2^)	28.1 ± 5.3	28.6 ± 5.4	28.5 ± 5.4	27.8 ± 5.0	27.7 ± 5.2	<0.001
SBP (mmHg)	125.6 ± 20.9	125.8 ± 21.2	124.9 ± 20.2	126.1 ± 20.5	125.5 ± 21.8	0.497
DBP (mmHg)	71.7 ± 10.2	71.8 ± 10.4	71.7 ± 10.0	72.0 ± 9.7	71.1 ± 10.5	0.135
FPG (mg/dl)	95.8 ± 27.4	93.8 ± 24.7	95.5 ± 27.9	97.0 ± 28.1	97.0 ± 28.6	0.009
TG (mg/dl)	110 (77–158)	113 (79–162)	112 (78–158)	107 (77–158)	107 (75–156)	0.183
TC (mg/dl)	193.5 ± 34.1	195.2 ± 34.4	194.2 ± 36.5	193.7 ± 33.1	191.0 ± 32.1	0.013
LDL-C (mg/dl)	117.1 ± 30.9	118.4 ± 31.2	118.2 ± 31.6	117.1 ± 30.7	114.7 ± 29.9	0.007
HDL-C (mg/dl)	51.3 ± 14.9	51.3 ± 15.2	50.8 ± 14.6	51.4 ± 14.8	51.8 ± 14.9	0.280
Homocysteine (μmol/L)	9.2 ± 3.7	9.5 ± 3.4	9.3 ± 4.3	9.1 ± 3.9	8.9 ± 2.9	<0.001
ARBs [n (%)]	278 (5.3%)	70 (5.3%)	62 (4.7%)	64 (4.9%)	82 (6.2%)	0.294
ACEIs [n (%)]	620 (11.8%)	157 (11.9%)	154 (11.7%)	160 (12.2%)	149 (11.3%)	0.925
Statins [n (%)]	778 (14.8%)	161 (12.2%)	186 (14.1%)	204 (15.5%)	227 (17.3%)	0.003
Unadjusted dietary vitamin B3 intake (mg/day)	16.2 ± 8.0	14.4 ± 7.1	16.0 ± 7.4	16.7 ± 8.3	17.7 ± 8.8	<0.001
Total energy intake (kcal/day)	1,527 ± 783.0	1823 ± 891.4	1,606 ± 752.7	1,450.5 ± 727.5	1,228.1 ± 611.7	<0.001

Values are presented as mean ± standard deviation or median (25th–75th quartiles) for continuous variables and n (%) for categorical variables. eGFR, estimate glomerular filtration rate; BMI, body mass index; SBP, systolic blood pressure; DBP, diastolic blood pressure; FPG, fasting plasma glucose; TG, triglycerides; TC, total cholesterol; LDL-C, low-density lipoprotein cholesterol; HDL-C, high-density lipoprotein cholesterol; ARBs, angiotensin receptor blockers; ACEIs, angiotensin-converting enzyme inhibitors.

After an average follow-up of 3.2 years, 1,261 participants exhibited renal function decline, 634 (20.9%) for those <65 years old and 627 (28.1%) for those ≥65 years old. As for participants ≥65 years old, highest dietary vitamin B3 intake group showed a reduced risk of renal function decline compared to the lowest group (Model 1: RR 0.744, 95%CI 0.574-0.964). The results did not appreciably change after adjustment of covariates (Model 2: RR 0.744, 95%CI 0.567-0.975; Model 3: RR 0.728, 95%CI 0.548–0.966). But this correlation could not be seen in the younger group with age <65 years old (Table 2). These finding suggests that high dietary vitamin B3 intake is associated with lower risk of renal function decline in the old population ≥65 years old.

**TABLE 2:** Risk of renal function decline for adjusted dietary vitamin B3 intake quartile groups.

	Decline/Total	Model 1, RR (95%CI)	*p*-Value	Model 2, RR (95%CI)	*p*-Value	Model 3, RR (95%CI)	*p*-Value
Age<65 group							
1	147/752	References	1.0	References	1.0	References	1.0
2	163/769	1.107 (0.862–1.421)	0.425	1.156 (0.896–1.493)	0.265	1.105 (0.851–1.436)	0.452
3	149/751	1.019 (0.790–1.314)	0.887	1.033 (0.794–1.344)	0.812	0.988 (0.752–1.298)	0.931
4	175/758	1.235 (0.965–1.582)	0.093	1.183 (0.911–1.536)	0.207	1.082 (0.822–1.424)	0.574
Age≥65 group							
1	180/563	References	1.0	References	1.0	References	1.0
2	146/546	0.777 (0.599–1.007)	0.056	0.783 (0.601–1.021)	0.071	0.771 (0.589–1.010)	0.059
3	157/564	0.821 (0.636–1.060)	0.130	0.800 (0.613–1.043)	0.099	0.783 (0.597–1.028)	0.078
4	144/556	0.744 (0.574–0.964)	0.025	0.744 (0.567–0.975)	0.032	0.728 (0.548–0.966)	0.028

## Discussion

In this study, we demonstrated that SIRT6 overexpression mitigated the renal interstitial fibrosis and renal function deterioration in CKD. HIPK2 was induced during the fibrogenesis, which was blocked after SIRT6 overexpression. SIRT6 depletion exacerbated the EMT of renal tubular epithelial cells, together with HIPK2 upregulation. HIPK2 was downregulated by SIRT6 in the post-transcriptional level. Population study indicated that old people with high dietary vitamin B3 intake tended to have lower risk of renal function decline. Taken together, our findings suggest that SIRT6/HIPK2 axis is a potential target to intervene renal fibrosis and delay CKD progression.

Renal fibrosis, mainly referred to renal interstitial fibrosis, is the inevitably common outcome of CKD progression. However, effective intervention to retard fibrotic process is still unavailable (Humphreys, 2018). As a longevity gene, SIRT6 presents a favorable prospect in fibrosis prevention (Yang et al., 2021). Indeed, SIRT6 is demonstrated to protect against liver fibrosis by inactivating hepatic stellate cells (Zhong et al., 2020; Zhang et al., 2021). Diabetic myocardial fibrosis is alleviated through SIRT6/AMPK signaling pathway (Li et al., 2020). SIRT6 deficiency also accounts for the aging-related cardiac fibrosis (Pillai et al., 2021). Previous study suggests that loss of SIRT6 aggravates unilateral ureteral obstruction-induced tubulointerstitial inflammation and fibrosis (Cai et al., 2020; Jin et al., 2022). While our study verified that renal interstitial fibrosis in adenine-induced CKD was alleviated by SIRT6 overexpression. And renal function was improved a lot after SIRT6 overexpression in CKD. Interestingly, SIRT6 was upregulated during renal fibrogenesis. Since improvement in renal fibrosis can also be seen through further SIRT6 overexpression, we thought it might be a compensatory mechanism. Besides, the level of NAD was declined in the development of CKD, which might also contributed to the defective function of SIRT6 (Liu et al., 2021).

Tubular epithelial cell undergoing epithelial-to-mesenchymal transition is recognized as a key contributor to renal fibrosis (Lovisa et al., 2016). While blunting this transition is considered as a promising strategy to reduce fibrogenesis (Xavier et al., 2015). Here, we found SIRT6 deficiency exacerbated epithelial-to-mesenchymal transition of tubular epithelial cells induced by TGF-β1. Likewise, SIRT6 mitigates kidney ischemia/reperfusion injury through retarding hypoxia-induced phenotypic transition of tubular epithelial cells (Gao et al., 2020). Other study also supports the powerful effect of SIRT6 on suppression of phenotypic transition. SIRT6 prevents pulmonary fibrosis by inhibiting pulmonary epithelial to mesenchymal transition (Tian et al., 2017; Chen et al., 2019).

Another important finding in our research was the suppression of SIRT6 on HIPK2. HIPK2 is a common upstream of multiple pro-fibrosis signaling pathway during renal fibrogenesis (Fan et al., 2014). Following study also finds that knockdown of HIPK2 is capable of alleviating Angiotensin II-induced cardiac fibrosis (Xu et al., 2022). Liver fibrosis is also mediated by HIPK2 activation in hepatic stellate cells (He et al., 2017). In our study, upregulation of HIPK2 in CKD-related renal interstitial fibrosis was blocked by SIRT6 overexpression. SIRT6 depletion accelerated the epithelial-to-mesenchymal transition of tubular epithelial cells, which was also associated with elevated HIPK2. Further study suggested that HIPK2 was inhibited by SIRT6 in post-transcriptional way. Indeed, deacetylation of HIPK2 is linked with its protein instability and subsequent proteasomal degradation (Hwang et al., 2013; Choi et al., 2017). Even though, whether HIPK2 is directly deacetylated by SIRT6 remains to be elucidated.

The activity of SIRT6 was dependent on the level of NAD. NAD biosynthesis is interrupted in response to kidney injury, while NAD-replacement therapy is considered to protect against human diseases (Bignon et al., 2022). Indeed, the precursors of NAD such as vitamin B3 or the simpler forms of vitamin B3 like nicotinamide or nicotinic are demonstrated to impede renal diseases (Streja et al., 2015; Hyndman and Griffin, 2021). Nicotinamide reduces renal interstitial fibrosis in mice (Zheng et al., 2019). Long-time treatment with vitamin B3 in CKD patients is capable of lowing serum phosphorous concentrations (Müller et al., 2007; Maccubbin et al., 2010; Malhotra et al., 2018). Here, we found that higher dietary vitamin B3 intake was related to slower renal function decline in the old population, but not the younger group. This might be attributed to that the NAD levels decline with aging, especially in the old people (Katsyuba et al., 2020). Vitamin B3 is known to increase NAD level in tissue, inhibit inflammation and oxidative stress, as well as improve lipid metabolism, which may account for its benefits in renal function (Wu et al., 2012; Ganji et al., 2015; Montserrat-de la Paz et al., 2019; Horimatsu et al., 2020). Even though, the limitation should be noticed that other nutriment intake was not considered in this study, which might also contribute to confounding.

## Conclusion

In summary, we uncovered the beneficial effect of SIRT6 overexpression on renal interstitial fibrosis of CKD, which might be associated with HIPK2 downregulation. We also highlighted the benefit of dietary vitamin B3 intake in the renal function of old population. SIRT6/HIPK2 axis may be a therapeutic target in the CKD treatment.

## Data Availability

The datasets presented in this study can be found in online repositories. The names of the repository/repositories and accession number(s) can be found in the article/Supplementary Material.
