# Supplementary figures and images for: SIRT6 overexpression retards renal interstitial fibrosis through targeting HIPK2 in chronic kidney disease
Source: Front Pharmacol. 2022 Sep 12;13:1007168. doi: 10.3389/fphar.2022.1007168 (PMC9510922; doi:10.3389/fphar.2022.1007168)

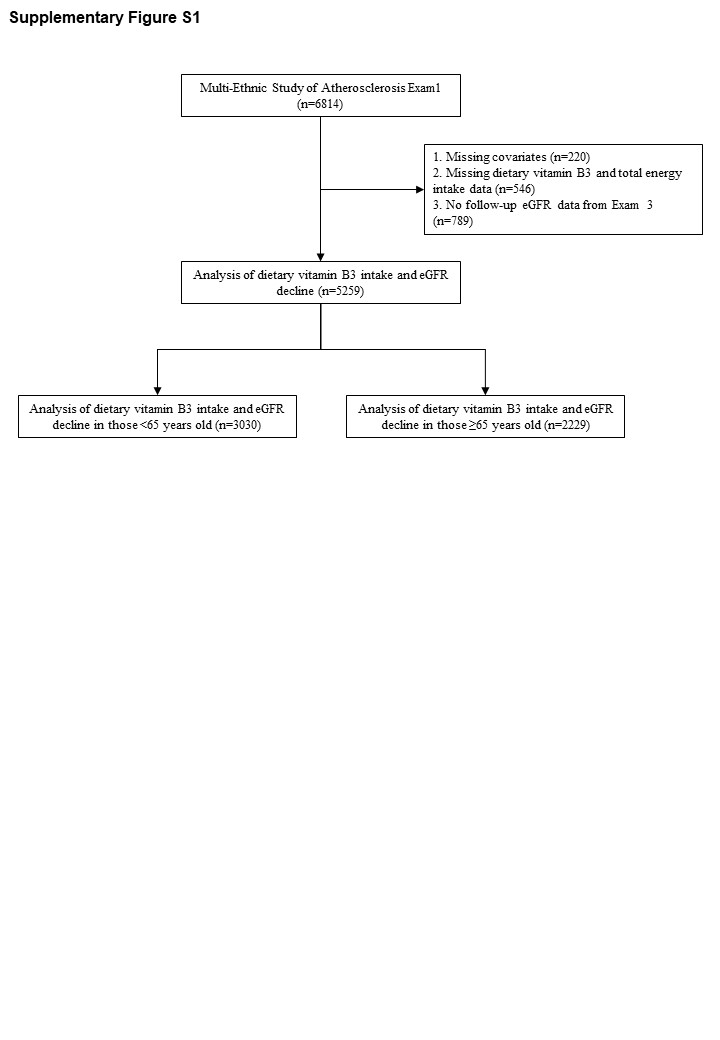

Supplement: Supplementary file 1 [file Image1.JPEG]
